# Lactoferrin: A Glycoprotein Involved in Immunomodulation, Anticancer, and Antimicrobial Processes

**DOI:** 10.3390/molecules26010205

**Published:** 2021-01-03

**Authors:** Quintín Rascón-Cruz, Edward A. Espinoza-Sánchez, Tania S. Siqueiros-Cendón, Sayuri I. Nakamura-Bencomo, Sigifredo Arévalo-Gallegos, Blanca F. Iglesias-Figueroa

**Affiliations:** Laboratorio de Biotecnología I, Facultad de Ciencias Químicas, Universidad Autónoma de Chihuahua, Circuito Universitario s/n Campus Universitario 2, Chihuahua C.P. 31125, Mexico; qrascon@uach.mx (Q.R.-C.); eaespinoza@uach.mx (E.A.E.-S.); tsiqueiros@uach.mx (T.S.S.-C.); sayu-nakamura@hotmail.com (S.I.N.-B.); sareval@uach.mx (S.A.-G.)

**Keywords:** lactoferrin, immune system, anti-cancer activity, antibacterial activity

## Abstract

Lactoferrin is an iron binding glycoprotein with multiple roles in the body. Its participation in apoptotic processes in cancer cells, its ability to modulate various reactions of the immune system, and its activity against a broad spectrum of pathogenic microorganisms, including respiratory viruses, have made it a protein of broad interest in pharmaceutical and food research and industry. In this review, we have focused on describing the most important functions of lactoferrin and the possible mechanisms of action that lead to its function.

## 1. Introduction

Current lifestyles and emerging diseases that threaten everyday life have led to the search for new, more natural, pharmacological alternatives to face new challenges in medicine. Various human-origin proteins have been studied for a long time because of their multifunctional characteristics in the body and their practical use as therapeutic agents. In this context, lactoferrin (Lf), described in the first reports as the “red fraction of the milk”, is a non-hematic iron-binding glycoprotein, secreted mainly by epithelial cells of the mammary gland [1,2]. According to its conserved three-dimensional structure and its ability to chelate iron ions, Lf belongs to the transferrin family [3] and has been reported as a nutraceutical and multifunctional glycoprotein for its antimicrobial, antiviral, and antifungal effects, and recently, it has been shown to be effective in the treatment of neuropathies and cancer cells [4]. In the present review, we focus on detailing the most important functions of lactoferrin, which make it highly attractive to the pharmaceutical industry.

## 2. Lactoferrin Structure and Production

**Figure 1:**
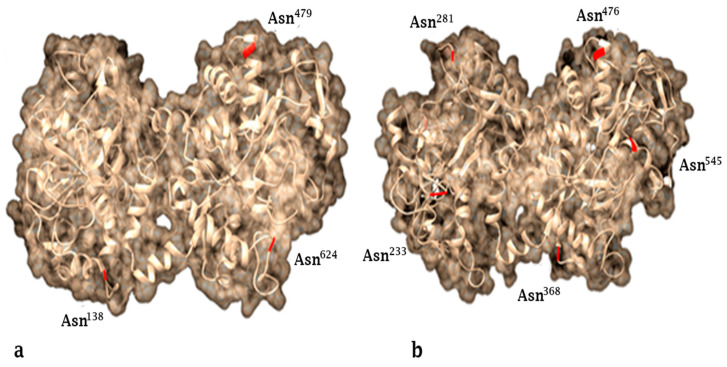
Predicted structure of Lactoferrin and its potential glycosylation sites (Asn^n^). (**a**) Human lactoferrin. (**b**) Bovine lactoferrin. Potential asparagine-linked glycosylation sites are shown in red. The glycosylation of the molecule may be strongly involved in the mechanism of action of its various physiological processes. The protein sequence was extracted from GenBank: M83202_1 (hLf) and M63502_1 (bLf) and modeled using Phyre2 (http://www.sbg.bio.ic.ac.uk/phyre2/ [18]).

Lactoferrin is a non-hematic iron-binding glycoprotein with a molecular weight of 78–80 kDa depending on the species. Lf is composed of a simple polypeptide chain with approximately 700 amino acids folded into two globular carboxyl (C) and amino (N) terminal lobes, which are regions connected through an α-helix and made up of two domains known as C1, C2, N1 and N2, which create a β-sheet [5,6,7]. The N-terminal lobe includes 1–332, amino acids, whereas the C-terminal lobe includes the amino acids 344–703 [8]. Three potential glycosylation sites have been found in human lactoferrin (hLf) (Asn138, Asn479 and Asn624) and five potential sites in bovine lactoferrin (bLf) (Asn233, Asn281, Asn368, Asn476 and Asn545) (Figure 1); these sites are mostly exposed on the outer surface of the molecule and may participate in recognition of specific receptors [9]. Lf has a high affinity for iron; each lobe can bind to a ferric ion; furthermore, it can bind Cu^+2^, Zn^+2^ and Mn^+2^ ions [10]. Mammals produce Lf, and its production is attributed to the cells of the epithelial mucosa within the majority of exocrine fluids, including tears, saliva, vaginal and seminal fluids, nasal and bronchial secretions, and bile and gastric juices. However, its highest concentration is found in milk and colostrum; humans produce approximately 2 g/L and 7 g/L in milk and colostrum, respectively, while in cows its concentration in milk and colostrum is 0.2 g/L and 1.5 g/L, respectively [11,12]. A considerable amount of Lf is found in the secondary granules of neutrophils (15 µg/106 neutrophils, and Lf is released into plasma during an inflammatory or infectious process. Under normal conditions, the concentration of Lf in plasma is 0.4–2.0 µg/mL and it increases up to 200 µg/mL in infections and immunological disorders [13,14,15]. The concentration of Lf in plasma is not related to the number of neutrophils, but depends on the degree of degranulation of these. Likewise, plasma Lf levels can be altered during pregnancy [16]. Lf has a high similarity between species; human Lf (hLf) and bovine Lf (bLf) show the highest degree of similarity in terms of structure and function; 78% of the human Lf sequence is identical to bovine Lf [17].

## 3. Immunomodulatory and Anti-Inflammatory Activity

**Figure 2:**
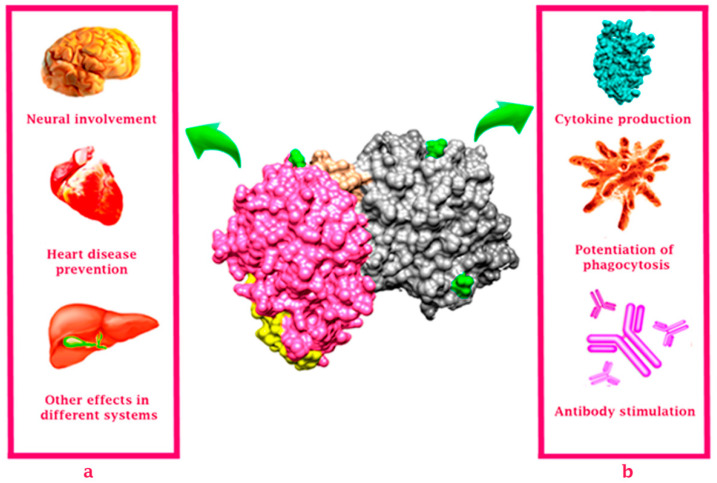
Schematic representation of the effects of lactoferrin in the body. (**a**) Non-dependent pathogenesis of Lf activities; Lf has implications on neurodevelopment and some neurodegenerative injuries and may be involved in the prevention of heart disease due to its effect on levels of lipoprotein accumulation. It can exert effects on metabolic activity in different systems. (**b**) Dependent pathogenesis of Lf activities; Lf can promote cytokine production, enhance phagocytosis and stimulate antibody production and various signaling pathways, in response to diverse diseases such as infection or cancer. Possible glycosylation sites are shown in green (Asn-138, Asn-479 and Asn-624), and the lactoferricin peptide is shown in yellow (amino acids 17–41). The N-terminal lobe (amino acids 1–332) is shown in pink and the C-terminal lobe (amino acids 344–703) is shown in gray.

The immunomodulatory and anti-inflammatory activity of lactoferrin is related to its ability to interact with specific cell surface receptors on epithelial cells and cells of the immune system, as well as its ability to bind to pathogen-associated molecular patterns (PAMPs), mainly recognized by Toll-like receptors (TLRs) [19]. Such binding has been reported for Gram-negative bacterial lipopolysaccharide (LPS) [20]. The mechanisms of the interaction of lactoferrin with various receptors are strongly linked to its glycan conformation; it has been observed that there is an interaction between some TLRs and Lf, mediated by glycans of the molecule, allowing an immunomodulatory effect [21,22]. Lf also plays a role in the differentiation, maturation, activation, migration, proliferation, and function of cells belonging to antigen-presenting cells (APCs), such as B cells, neutrophils, monocytes/macrophages, and dendritic cells [23,24]. In vitro and in vivo studies have shown that macrophages and dendritic cells are capable of binding Lf through its interaction with surface receptors for Lf that induce its maturation and, therefore, its functional activity [25,26,27]; in addition, the effect of Lf on the differentiation and activation of monocytes/macrophages contributes to reducing the pro-inflammatory profile [28]. On the other hand, Lf can also reduce the inflammatory response in a diversity of pathologies. In allergic rhinitis, it achieves this by regulating the function of Th1 and Th2 cells, promoting the Th1 response through the synthesis of IL-2 and IFN-γ and inhibiting the Th2 response, reducing the release of inflammatory mediators such as IL- 5 and IL-17 and causing the crosslinking of the T cell receptor, so that the activation of T cells is inhibited [29]. In colitis, Lf promotes the reduction of various inflammatory mediators such as TNF, as well as the infiltration of CD4 cells, helping to improve the inflammatory state [30]. In these contexts, Lf administration has also been shown to contribute to mucosal repair during Crohn’s disease [31]. A recent study showed that Lf could counteract the novel coronavirus infection and inflammation by acting as a natural barrier, reversing iron disorders related to viral colonization and modulating the immune response by down-regulating pro-inflammatory cytokines [32], preventing a cytokine storm from being generated, a condition that can aggravate the prognosis of diabetic patients with Covid-19 [33]. Taken together, the immunomodulatory actions triggered by Lf may intervene in different organs and systems on which a lactoferrin-mediated effect has been observed (Figure 2).

## 4. Iron-Mediated Lactoferrin in Neuropathies

All neuropathies together have a prevalence of more than 2% in the general population, but a prevalence of greater than 15% in those over the age of 40 [34,35,36]. Moreover, the prevalence of chronic neuropathies, which are progressive and commonly age-associated, such as Alzheimer’s disease, Huntington’s disease, multiple sclerosis, transmissible spongiform encephalopathies and Parkinson disease, has been increasing in recent years, representing a considerable challenge for societies [37,38]. There is no cure for any of these diseases and the approved medications are ineffective or not tolerated by many patients. Therefore, the current treatments are focused on the control of symptoms [34,39,40]. Because the regulation of iron deposits is critical to efficient management of neural cells, several mechanisms are used by the organism to decrease iron-related stress such as neuromelanine synthesis, transferrin transport, iron regulation, mitochondrial iron sequestration, and heme oxygenase-1 (HO-1) induction [41,42,43,44]. In addition, microglia cells that exhibit Lf that is sialic acid-rich with a high iron-binding capacity [45,46] have been associated with early neurodevelopment and cognitive function in mammals, an increase in cellular protrusions, microtubule dynamics, formation and organization of neurite outgrowth, cytoskeleton formation, and a decrease in anxiety [47,48,49]. It was observed that when the Lf is attached to iron, it prevents spontaneous and progressive death of dopaminergic (DA) neurons. In addition, it can prevent the death of a large neuron population that is already damaged [46]. On the other hand, it has been suggested that the protective effect of Lf against the spontaneous loss of DA neurons may possibly result from an indirect effect on dividing glial cells [50,51] because treatments with Lf can increase the division of microglial cells, which are important mediators in inflammatory processes and have a neuroprotective function in the brain [52,53]. As well as the iron-binding capacity, when the microglia are activated by a neurodegenerative process, Lf mRNA is increased, as well as their receptors in DA neurons [54,55]. Once the Lf is produced, it is retained in DA neurons where the proximal regions bind to heparan sulfate proteoglycans (HSPGs) [46]. It is possible that the protective effect of Lf in dopaminergic neurons is also due to direct competitive union in HSPGs. It is reported that Tau proteins bound to HSPGs trigger the aggregation of intracellular fibrils like prions that can drive the progression of Alzheimer’s disease, frontotemporal dementia and other tauopathies in a prion-like manner. Therefore, the interference of tau binding to HSPGs, mediated by the union of Lf, prevents recombinant tau fibrils that cause intracellular aggregation and blocks transcellular aggregate propagation and the subsequent neuropathology [56]. On other hand, assays using MPTP (1-methyl-4-phenyl-1,2,3,6-tetrahydropyridine) neurotoxin in DA neurons, which causes mitochondrial damage, have shown that the neuron death occurred by a decrease of Ca^2+^_mit_ levels and that the addition of Lf stimulated the phosphorylation of protein kinase B (P-AKT), producing a sustained rise in Ca^2+^_mit_ resulting in a robust increase in DA neuron cell survival [17,46,57]. These observations suggested that the protective effect of Lf is also due to its capacity to modulate the mitochondrial-Ca^2+^ mechanism controlled by phosphatidylinositol 3-kinase (PI3K), which stimulates Ca^2+^ mobilization in the endoplasmic reticulum [46]. Because Lf is implicated in the improvement in cognition and neural development, and can modify the progression of the degenerative process, the production of Lf might be of interest for the treatment of neurodegenerative diseases. Furthermore, because the plasma level of Lf is inversely correlated with disease severity, this might be evidence of an attempt by the brain to combat ongoing neuronal insults and may be useful as a neuropathy indicator [46,47,58,59,60].

## 5. Anticancer Activity

According to the World Health Organization, cancer is one of the leading causes of morbidity and mortality in developed and underdeveloped countries and is the second leading cause of death globally [61]. Current cancer treatments invariably involve physiological and psychological collateral damage. In addition, treatment with chemotherapy may have side effects on fertility and in the case of women, premature menopause, leading to an increased risk of osteoporosis. Furthermore, the most common treatments involve a risk of heart damage [62]. In this sense, researchers are searching for more natural anti-cancer treatments to decrease the collateral damage in oncological patients. The anticancer effects of Lf have been extensively studied, and it has been observed that in the presence of Lf, different cancer cells suffer significant damage, such as cell cycle arrest, damage to the cytoskeleton, and induction of apoptosis, in addition to a decrease in cell migration [63,64]. Even though this damage has been observed, the mechanism that underlies these effects remains to be elucidated. There are several possible mechanisms through which Lf can exert its anticancer effect; for one side, various authors have proposed that the basis of lactoferrin’s anticancer action could reside in cell signaling and recognition through the glycans that make up its structure [65]. On the other hand, it is known that many cancer cells have a high content of proteoglycan, glycosaminoglycan and sialic acid, which are known to interact with Lf, which probably activates other signaling pathways to generate harmful effects to cells [66]. This possible mechanism will also explain the high cytotoxic selectivity that Lf has on cancer cells and not on healthy cells [65,67,68,69,70]. Despite the fact that various authors showed that Lf has high selective cytotoxicity, not all reported the selective cytotoxicity index (SCI), which gives the ability of a compound to kill cancer cells with minimal toxicity to non-cancer cells. Table 1 shows the different SCI values for Lf against different types of cancer cells reported by our research group. Finally, iron metabolism is strongly involved in the metabolic requirements of some cancer cells. It may even lead to the metastasis of tumor cells [71], so that Lf, being a molecule capable of chelating iron ions, also has a mechanism of anti-cancer action based on its ability to balance this ion in the organism [72]. Here, we describe the effect of Lf on the most prevalent types of cancers worldwide.

### 5.1. Lactoferrin in Breast Cancer

The supply of estrogens represents a crucial factor in the development of most breast cancers. In the same way, iron homeostasis correlates with estrogen production, a decreased level of iron promotes angiogenesis, and superior levels of iron contribute to an increase in oxidative stress. A natural bridge between iron and estrogen is Lf [73]. As thyroid steroid receptors modulate the expression of the Lf gene, this gene is sensitive to hormones, so Lf may be involved in hormone dependent cancers, such as breast cancer, where its expression seems to be progressively inhibited [74]. On the other hand, in non-hormone dependent cancers like triple negative breast cancer, where hormone-targeted therapies are not available and the prognosis in general is not favorable [75], Lf could also be a potential alternative treatment as it has been shown to have an in vitro cytotoxic effect on human triple negative breast cancer cells. In this sense, we previously reported that recombinant human lactoferrin from *Pichia pastoris* has an apoptotic effect and causes cell cycle arrest in the S phase in non-metastatic and metastatic MDA-MB-231 cells [65]. This seems to extend to Lf from other species; bovine lactoferrin (bLf), both in its free-iron form and in its iron-saturated form, has been used successfully in the induction of cytotoxicity and the reduction in cell proliferation of MDA-MB-231 and MCF-7 human breast cancer cells [76,77]. In the same way, both forms of Lf can modulate some apoptotic molecules, including p53, and completely inhibit the expression of survivin, a multifunctional protein involved both in the apoptotic inhibition and in the regulation of the cell cycle, which promotes resistance to cancer cells in chemotherapy and radiotherapy [77,78]. On the other hand, it has been reported that treatment with nanoparticles of calcium phosphate loaded with bovine saturated Lf is able to decrease the size of the tumors in murine models [79]. Alternatively, specific bioactive peptides from Lf have also been used to test their antitumor effect, such as lobe C from hLf, which was used against breast cancer, promoting cellular apoptosis and generating significant growth arrest in MDA-MB-231 cells [80]. These data indicate that both bovine and human Lf has high efficacy in the control of tumor proliferation in breast cancer.

### 5.2. Lactoferrin in Leukemia

Of all cancer patients worldwide, only approximately 1% corresponds to childhood cancer. The control of this disease in children is limited. Leukemia is the cancer with the highest prevalence among children [81]. Currently, treatments for different types of leukemia, including acute myeloid leukemia and acute lymphoid leukemia, improve the survival rate in diagnosed children; however, the long-term consequences can manifest as cardiovascular diseases that increase the risk of death [82,83]. In this sense, once again, Lf could be an alternative that minimizes side effects in patients, since it was shown to induce apoptosis in leukemia [70,84]. In the same way that Lf has been successfully tested for the treatment of cancers, primarily breast cancer, both Lf and bioactive Lf-derived peptides have been used successfully in the treatment of other types of cancer such as leukemia. In addition, it was demonstrated that PRF peptide, a fragment of hLf, also has antitumor activity. This peptide showed induction of cell death in leukemia cells, causing a necrotic effect. Moreover, PRF peptide induced G0/G1 cell cycle arrest [85]. Another peptide from bLf, lactoferricin B, exerts a potent cytotoxic effect on Jurkat and CCRF-CEM T-leukemia cells [86,87]. Furthermore, it can increase caspase-3 expression, promoting DNA fragmentation and, therefore, the apoptosis pathway in the HL-60 leukemia cell line [88]. Finally, it has been shown that in infants, exclusive and prolonged consumption of breast milk can prevent the risk of developing childhood leukemia due to various immunoprotective agents present in breast milk, such as lactoferrin [89,90].

### 5.3. Lactoferrin in Cervical Cancer

Cervical cancer is one of the most prevalent types of cancer worldwide and the fourth leading cause of death in women [91]. It affects women from developing countries and a low socioeconomic level most due to the lack of access to preventive tests such as pap smears and vaccines against human papillomavirus (HPV), the causative agent of cervical cancer [92]. One of the most widely used therapies in this type of cancer is radiation therapy, by itself or in combination with other methods such as surgery; however, it is reported that the effect of radiotherapy could be more damaging than beneficial, as it can cause recurrence. In this context, the use of potential therapeutic agents such as Lf can decrease collateral risk in patients [93]. Again, the impact that Lf has on iron metabolism may be related to its anti-cancer mechanism, as previous studies showed that Apo-Lf, but not Holo-Lf, induced apoptosis in HeLa cells and modified the expression of proapoptotic proteins such as BAX, which was increased in the presence of Lf, unlike Bcl-2 and Mcl-2, which decreased its expression [94].

## 6. Antimicrobial Activity

Due to the multiple mechanisms that lactoferrin exerts, its activity against a wide spectrum of microorganisms that are pathogenic for humans has been well documented [95]. In this context, several mechanisms of action of Lf have been demonstrated against bacteria, fungi, parasites and viruses, including possible activity against the novel coronavirus SARS-CoV-2 infection [17,32]. Two of the principal mechanisms by which lactoferrin is capable of exerting its antimicrobial activity are related to its ability to sequester iron [96] and the direct action of its bioactive peptide, lactoferricin [97]. On the other hand, recent studies have shown that the molecule’s glycosylation status may be an important factor in enhancing its antimicrobial effect [98].

### 6.1. Antibacterial Activity

**Figure 3:**
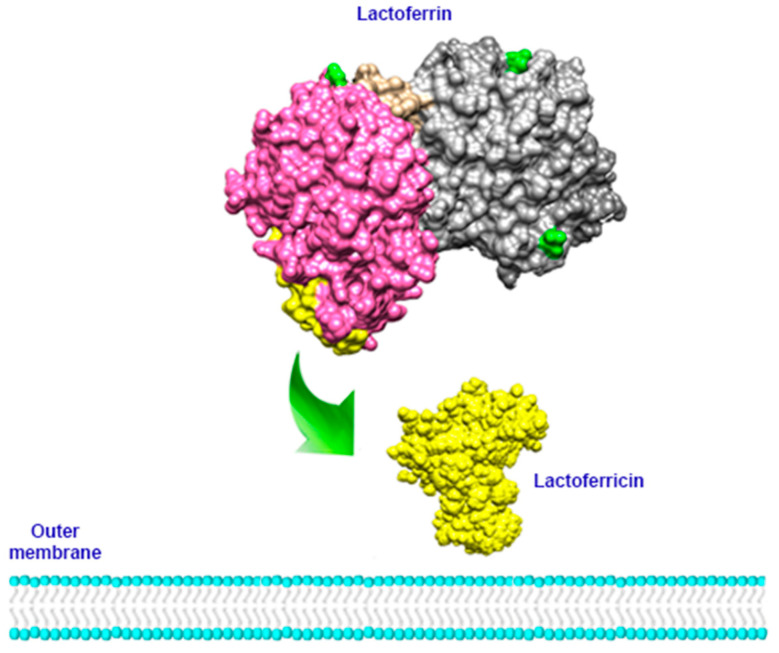
Schematic representation of Lfcin binding with LPS. When Lfcin (yellow) is released from Lf, it can bind to bacterial LPS, activate the immune response, and disrupt the bacterial outer membrane.

**Figure 4:**
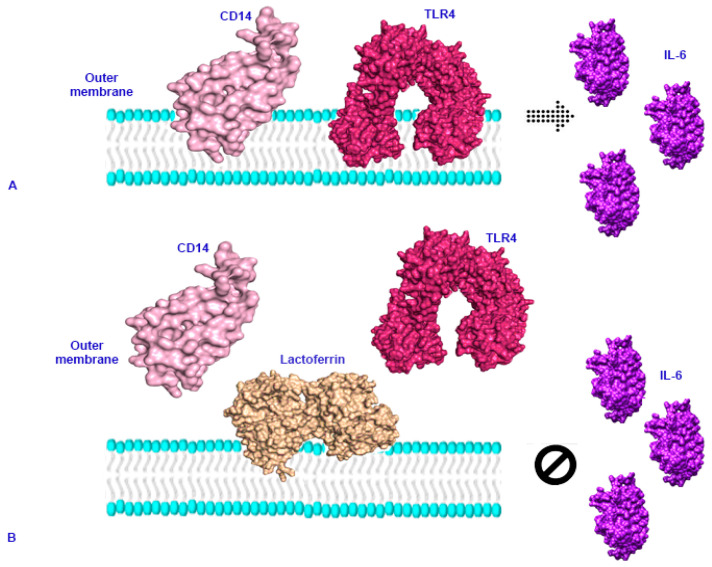
Schematic representation of the interaction of lactoferrin with LPS. (**A**) The interaction of LPS with CD14 can trigger an inflammatory response, with the release of some pro-inflammatory mediators such as IL-6 via TLR4. (**B**) In the presence of Lactoferrin, it can bind to LPS and block the interaction with CD14. Thus, the signaling of pro-inflammatory mediators through TLR4 can be diminished. The protein sequence was extracted from GenBank: CAG33297 (CD14), AF177765.1 (TLR4), and AAD13886.1 (IL-6).

In recent years, the antibacterial effect of Lf has been reported in vivo and in vitro; Lf exerts a bacteriostatic and bactericidal effect on Gram negative bacteria such as *E. coli, Ps. aeuruginosa*, *Salmonella*, *Enterobacter*, *H. pylori*, *Yersinia*, *Klebsiella pneumoniae*, and *Porphyromonas gingivalis*, [97,99,100,101,102,103,104,105,106] and on Gram positive bacteria such as *Bacillus, Listeria monocytogenes* and *S. aureus* [99,106,107]. It has various mechanisms of action. On the one hand, it works through its bioactive peptide, lactoferricin, which is capable of destabilizing the bacterial membrane (Figure 3) and, consequently, increasing its permeability, allowing the passage of other antibacterial substances such as lysozyme, which enhances the bactericidal effect. In addition, Lf is able to compete with LPS for the binding of CD14; this binding prevents LPS from unleashing the production of pro-inflammatory cytokines that in turn lead to tissue damage in the host (Figure 4) [108,109]. On the other hand, in Gram-positive bacteria, Lf can bind to the lipoteichoic acid of the cell wall, again promoting a destabilization of the membrane and together with lysozyme, it generates a bactericidal effect [8]. In addition, the presence of Lf can improve the effect of certain antibiotics such as levofloxacin, rifampicin, clarithromycin, and clindamycin against various pathological agents, which suggests that the use of this molecule can give a significant boost to current treatments against different diseases [104,110].

### 6.2. Antiviral Activity

The antiviral activity of Lf has been observed against enveloped and naked viruses, such as influenza virus (H1H1, H3N2), human norovirus, human papillomavirus, Chikungunya, Zika, and HIV [111,112,113,114,115,116]. Furthermore, Lf could have a preventive role in SARS-CoV-2 infection, due to its observed effect on the internalization of SARS-CoV-1, in addition to its ability to decrease the inflammatory response [117]. Some studies have shown that lactoferrin is able to inhibit infection by pseudovirus SARS [118]. In this sense, it is believed that breast milk, which contains significant amounts of Lf, can confer important protection against the new coronavirus SARS-CoV-2 on the newborn [119]; unfortunately, many studies are still needed to confirm the behavior of the novel coronavirus and its subsequent treatment, but Lf seems to be a very promising prophylactic option. Its antiviral activity resides in its ability to block certain receptors, such as heparan sulfate glycosaminoglycan cell receptors, and by interacting with viral hemagglutinin, which can make Lf capable of breaking the viral envelope [120,121,122]. A key to understanding these interactions could be within its glycosylation profile; some reports showed how an alteration in the glycosylation of the molecule could, in turn, alter the signaling intensity of various toll-like receptors, like TLR-3 and TLR-8, involved in the recognition of viral particles [123,124]. However, there are still many questions to be answered to have a clearer picture of the glycoprotein antiviral activity.

### 6.3. Antifungal Activity

The high prevalence of vulvovaginitis caused by *Candida* around the world is an important public health problem due to the rise in medical costs and the mortality rate in immunocompromised patients [125]. Lf, together with lactoferricin, lactoferrampin and the N-terminal region (Lf 1-11), has shown activity against different species of *Candida* by altering its cell wall and generating surface blebs, which cause cell death [126,127]. Likewise, Lf has been used in synergy with different antifungal drugs against different yeasts such as *C. dubliniensis, C. albicans, C. glabrata,* and *Cryptococcus*, where their effect is enhanced [128,129]. In the same context, Lf activity against *Candida albicans* was also enhanced when it was expressed in *Lactobacillus casei*, a member of the vaginal microbiota [130]. Other fungi such as *Aspergillus nidulans* have been susceptible to treatment with Lf [131]. Therefore, Lf has been observed to have well-defined antifungal capacities, which opens the opportunity for new lines of research in this field.

### 6.4. Antiparasitic Activity

Although the antimicrobial effect of Lf against viruses, bacteria and fungi has been extensively studied, its study in parasites has been much more difficult due to the complexity of these organisms. However, current lines of research suggest that lactoferrin may be internalized by receptor-mediated endocytosis, producing irreversible cell damage [132], in addition to the effect of Lf on the balance of T cells, which may contribute to the response to this type of infection [133]. These antiparasitic effects of Lf and its bioactive peptides have been observed in different organisms such as *Giardia lamblia*, *Entamoeba histolytica* and *Trypanosoma* [132,133,134,135]. On the other hand, Lf from different species has been nanoencapsulated to test its effectiveness against various parasite species. This treatment has been successful against *Plasmodium berghei* and *Toxoplasma gondii* [136,137].

### 6.5. Antimicrobial Activity in Domestic Animals

Finally, the use of Lf as a protective molecule against pathogenic microorganisms is not limited to humans. Several studies have proven the beneficial activity of Lf against pathogenic microorganisms of veterinary importance such as *Babesia caballi*, the protozoan that causes equine babesiosis [138]; further, its co-administration with antibiotics such as penicillin can help to cure mastitis in cattle caused by *S. aureus*, *Streptococcus uberis* and *Streptococcus dysgalactiae* [95]. On the other hand, the protective effect of Lf has been tested in weaned piglets. When piglets were treated with Lf, the population of *Salmonella* and *E. coli* was reduced, and beneficial bacteria such as *Lactobacillus* and *Bifidobacterium* increased because of Lf [139]. In this sense, Lf also contributes to increasing the growth performance of recently weaned piglets by reducing the incidence of diarrhea, most likely due to its immunoprotective and antimicrobial effects [140,141].

## 7. Other Applications of Lactoferrin

Because of its wide distribution in the body, Lf may be involved in pivotal roles in various organs and systems. Studies in vivo have described its beneficial effect in bone regeneration processes [142,143], and on the prevention of metabolic diseases such as obesity and diabetes [144], which would have a positive global impact on the entire body.

## 8. Concluding Remarks

Despite the extensive literature that supports the contributions of lactoferrin in health and disease, various mechanisms of action of the protein remain the subject of research. Our research group has published several reports focused on the anti-cancer and antimicrobial activity of Lf. These reports have addressed the interactions of the different possible glycoforms of different species of Lf with cellular receptors. The understanding of the mechanism of action of Lf in different pathologies is still not entirely clear. Some obstacles must be resolved, such as the interactions between it and different cell receptors, its mechanisms of action, the repercussions of in vivo treatments, and the degree that the structural differences of the diverse lactoferrins effect the treatments. However, it is a research field that is worth exploring, because it can contribute to current treatments against highly important pathologies worldwide.

## Figures and Tables

**Table 1:** Selective cytotoxicity index (SCI) of lactoferrin against different cancer cells.

Cell Line	Cancer Type	SCI	Ref.
MDA-MB-231	Human triple negative breast cancer MDA-MB-231 cell line; non metastatic	11.68	[65]
MDA-MB-231-LM2-4	Lung metastatic (LM) variant derived from MDA-MB-231 cells	13.99	[65]
CCRF-CEM	Peripheral blood-derived leukemia cells, from a 4-year-old female	23.25	[70]
HeLa	Tumor’s epithelial cells derived from an adult with cervical adenocarcinoma	9.59	[70]
Sup-T1	T-lymphoblast from an 8-year-old male with T-cell lymphoblastic lymphoma	6.12	[70]
